# Family history of diabetes is associated with diabetic foot complications in type 2 diabetes

**DOI:** 10.1038/s41598-020-74071-3

**Published:** 2020-10-13

**Authors:** Xiao-fen Xiong, Ling Wei, Ying Xiao, Ya-Chun Han, Jinfei Yang, Hao Zhao, Ming Yang, Lin Sun

**Affiliations:** Department of Nephrology, The Second Xiangya Hospital at Central South University, Hunan Key Laboratory of Kidney Disease and Blood Purification, No.139 Renmin Middle Road, Changsha, 410011 Hunan China

**Keywords:** Endocrine system and metabolic diseases, Diabetes, Diabetes complications

## Abstract

To investigate the relationship between diabetic foot complications (DFCs) and clinical characteristics, especially the number and types of first-degree family members with diabetes. A total of 8909 type 2 diabetes patients were enrolled. The clinical characteristics of these patients, including DFCs and family history of diabetes (FHD), were collected from medical records. Multiple regression was used to investigate the association between FHD and DFCs after adjusting for confounding factors. The patients with one and more than one first-degree family member with diabetes accounted for 18.7% and 12.8%, respectively. The proportions of the participants with a father with diabetes, a mother with diabetes, both parents with diabetes, siblings with diabetes, father and siblings with diabetes, mother and siblings with diabetes, and both parents and siblings with diabetes were 3.5%, 6.2%, 1.1%, 14.4%, 1.5%, 4%, and 0.7%, respectively. The multiple regression analysis showed that the number of family members with diabetes was positively associated with DFCs. However, among the different types of FHD, only the patients with a mother with diabetes showed a statistical association with DFCs. In addition to FHD, other factors, including gender, body mass index, platelet count, hemoglobin levels, albumin levels, high-density cholesterol levels, diabetic peripheral neuropathy, and the use of lipid-lowering agents, oral hypoglycemic agents, and insulin, were also associated with DFCs. DFCs were associated with different numbers of family members with diabetes and types of FHD. This association reveals the importance of genetic and environmental factors in DFCs and highlights the importance of adding FHD to public health strategies targeting detecting and preventing the disease.

## Introduction

Globally, the number of people living with diabetes mellitus (DM) has increased fourfold over the past 30 years^1^. Type 2 diabetes mellitus (T2DM), accounting for 90% of cases of diabetes, leads to diabetes-related complications, such as microvascular and macrovascular complications, which impair patients’ physiological and psychological well-being and impose a considerable burden on the global health-care system^2^.

Among the diabetes-related complications, diabetic foot complications (DFCs), a condition of chronic ulcer, occur in 25% of diabetic patients^3^ and cause nontraumatic lower-extremity amputations (LEAs) worldwide^4^. Although there is a decreasing trend in the annual incidence of LEAs caused by T2DM^5^, the cost of ulceration and amputations attributable to diabetes reached $10.9 billion in 2001^6^. Several risk factors are correlated with DFCs, such as severe peripheral arterial disease (PAD)^7^, poor glycemic control and neuropathy^8^, and foot deformity^9^. A prospective study with a follow-up period of 10 years observed that the risk of first major amputation for DFC patients with PAD was 35 times higher than the risk for those without PAD^7^. However, it remains unknown whether other risk factors, including family history of diabetes (FHD), especially the different number and different types of first-degree family members, and other clinical characteristics are associated with DFCs.

FHD, an easily attended and significant medical record in the clinic, has been observed to have a significant association with diabetes^10–13^. A previous study observed a fourfold increase in the risk of T2DM in participants with FHD compared with those without FHD^14^. Additionally, FHD is closely related to diabetes-related complications, such as diabetic retinopathy (DR)^15,16^ and neuropathy^17^. A study focused on parental history of diabetes and DR found that the patients with a parental history of diabetes indicated a high prevalence of DR even after adjusting for usual confounders of DR^15^. However, few studies have concentrated on the relationship between DFCs and FHD, especially of the different number and different types of first-degree family member with diabetes.

Therefore, the current study aimed to investigate the relationship between DFCs and clinical characteristics, especially of the different numbers (such as one or more) and different types (such as mother, father, or siblings) of first-degree family members with diabetes.

## Methods

### Study population

A total of 8909 T2DM patients from the National Clinical Research Center for Metabolic Diseases Diabetes Center, P.R. China, the Department of Endocrinology of the Second Xiangya Hospital of Central South University were recruited for the study from January 2013 to November 2018. All clinical characteristics were collected from the patients’ medical records, including the FHD and DFCs. The inclusion criteria were as follows: (1) type 2 diabetes and (2) age of disease onset ≥ 18 years old. The exclusion criteria were as follows: (1) type 1 diabetes or latent autoimmune diabetes in adults (LADA); (2) pregnancy or lactation; (3) cancer; (4) secondary disease resulting in elevated blood glucose; (5) patients with missing values of FHD and DFCs. A detailed flow chart is shown in supporting information Table 1. The study was approved by the Hunan Research Ethics Committees in the Second Xiangya Hospital of Central South University, China. All participants in this study provided informed consent prior to their inclusion in the study. All methods were carried out in accordance with relevant guidelines and regulations.

The definition of diabetes was mainly based on the standards of the American Diabetes Association (ADA)^18^.

### Measurements

The measurement of hemoglobin (Hb) and platelet (PLT) levels were performed by the automated hematology analyzer ADVIA 2120 (Siemens Healthcare Diagnostics, Germany). The biochemical parameters, consisting of albumin (Alb), creatinine (Cr), uric acid (UA), fasting blood glucose (FBG), total cholesterol (TC), low-density lipoprotein-cholesterol (LDL-C), and high-density lipoprotein-cholesterol (HDL-C) levels, were evaluated by the Hitachi 912 automated analyzer. The standard methods and automatic high-performance liquid chromatography (VARIANT-II Hemoglobin Testing System; Bio-Rod Laboratories, Hercules, CA) were performed to measure the levels of fasting C-peptide and glycosylated hemoglobin (HbA_1C_), respectively. Additionally, the immunoturbidimetric method was used to measure proteinuria.

### Definitions

The definition of FHD was the presence or absence of diabetes among the first-degree family members, including father, mother, and siblings. According to the different types of first-degree family members with diabetes, all patients were subdivided into seven groups, including fathers with diabetes, mothers with diabetes, parents with diabetes, siblings with diabetes, fathers and siblings with diabetes, mothers and siblings with diabetes, or both parents and siblings with diabetes. The formula of the body mass index (BMI) and waist-hip ratio (WHR) were as follows: weight (kg)/height (m^2^) and waist/hip, respectively. FBG and fasting c-peptide levels were used to calculate homoeostatic model assessment 2 of β-cell function (HOMA2-B) and insulin resistance (HOMA2-IR). The estimated glomerular filtration rate (eGFR) was calculated based on the Modification of Diet in Renal Disease (MDRD) formula. The definition of drinkers or smokers was a drinking or smoking time ≥ 1 year. The treatment of angiotensin-converting enzyme inhibitor (ACEI), angiotensin receptor blockers (ARB), oral hypoglycemic agents (OHA), or insulin were referred to regular medications taking ≥ 3 months.

The diagnosis of DFCs was based on the following three criteria: (1) diagnosed with diabetes; (2) foot tissue dystrophy (ulcer or gangrene); and (3) accompanied by neuropathy or/and vasculopathy^18^. Neurologic examination was used to assess the foot dorsum and position in the toes sense and to evaluate the patellar reflexes and deep tendon. DR is an important manifestation of diabetic microvascular disease, which is classified as no proliferative diabetic retinopathy (NPDR) or proliferative diabetic retinopathy (PDR) based on new retinal blood vessel formation. Diabetic peripheral neuropathy (DPN) was diagnosed by clinical symptoms of sensory nerve and autonomic nerve (such as persistent pain and/or sensory disturbance in the extremities or lower extremities; decreased sensation), the examination of neurology and electrophysiologic investigations. Diabetic nephropathy (DN) was defined by a urinary albumin creatinine ratio (UACR) ≥ 30 mg/24 h. Microalbuminuria was defined as UACR ≥ 30 mg/24 h but ≤ 300 mg/24 h, and macroalbuminuria was defined as UACR ≥ 300 mg/24 h. The definition of coronary heart disease (CHD) was impaired myocardium attributable to imbalanced coronary blood flow and myocardial demands, including acute coronary syndrome and chronic coronary syndrome, as described in a previous study^19^. Cerebrovascular disease (CVD) is mainly caused by intracranial blood circulation disorders caused by brain vessels, such as stoke and cerebral infarction, as previously described^20^. Hypertension (HTN) was defined as the SBP greater than 130 mmHg or the DBP greater than 80 mmHg.

### Statistical analysis

Continuous variables are presented as the mean ± standard deviation, and discrete variables are described as percentages. For continuous variables, one-way ANOVA or the Kruskal–Wallis H test were used to compare the differences among groups. For discrete variables, we used the Chi-square test to compare groups. Multivariate regression analysis was used to estimate the association between DFCs and clinical variables. The variables selected for the multivariate regression analysis were based on the statistical significance of univariate regression analysis, including sex, DBP, durations, smoking, BMI, FHD, Hb levels, PLT levels, LDL-C levels, HDL-C levels, TC levels, Alb levels, HOMA-IR, eGFR, lipid-lowering agents, OHA and insulin (see supporting information Table 3), whereas disease onset age and HbA_1c_ and DPN were forcibly selected for the multivariate regression analysis. All variables in the regression analysis were described as positive or negative based on a cut-off number, which is described in detail in the supplementary materials (see supporting information Table 2). The analysis was performed by SPSS 25.0, and a two-tailed *P* value < 0.05 was considered statistically significant.

## Results

### The clinical characteristics of the participants

A total of 8909 participants diagnosed with T2DM were recruited for the study. The mean age of all patients was 57.89 ± 12.2 years, and the mean disease onset age was 49.33 ± 11.37 years. Men accounted for 55.4% of the sample. A total of 2804 (31.5%) patients had first-degree family members with diabetes. Among them, the patients with one and more than one first-degree family member with diabetes accounted for 18.7% and 12.8%, respectively. The proportions of the participants with a father with diabetes, a mother with diabetes, both parents with diabetes, siblings with diabetes, a father and siblings with diabetes, a mother and siblings with diabetes, and both parents and siblings with diabetes were 3.5%, 6.2%, 1.1%, 14.4%, 1.5%, 4%, and 0.7%, respectively. The mean concentrations of FBG and HbA_1c_ were 8.60 ± 4.02 mmol/l and 9.06% ± 2.39%, respectively. The patients with HOMA-IR ≥ 1 accounted for 48.4%. Moreover, among the diabetes-related complications, nearly half of the subjects had DPN (50.7%), and only a small proportion of patients had DFCs (7.7%). Additionally, regarding the treatments, a large proportion of people took lipid-lowering agents (71.3%), OHA (70.9%) and insulin (70.2%). Only 17.5% and 23.1% of participants took ACEIs and ARBs, respectively (see Table 1).

**Table 1:** Clinical characteristics of the participants.

Basic characteristics	
N	8909
Age (years)	57.89 ± 12.2
Men (%)	4947 (55.4%)
BMI (kg/m^2^)	24.62 ± 3.68
WHR	0.94 ± 0.07
SBP (mmHg)	137.45 ± 20.95
DBP (mmHg)	80.63 ± 12.18
Durations (years)	8.6 ± 6.7
Disease onset age (years)	49.33 ± 11.37
**Family history of diabetes**	
One family member with diabetes (%)	1663 (18.7%)
≥ 2 family members with diabetes (%)	1141 (12.8%)
Father (%)	313 (3.5%)
Mother (%)	555 (6.2%)
Both parents (%)	97 (1.1%)
Siblings (%)	1287 (14.4%)
Father and siblings (%)	137 (1.5%)
Mother and siblings (%)	354 (4.0%)
Both parents and siblings (%)	61 (0.7%)
Smoking (%)	3069 (34.4%)
Drinking (%)	2138 (24%)
**Laboratory data**	
Hb (g/L)	126.46 ± 21.77
PLT (*10^9^/L)	213.89 ± 81.64
FBG (mmol/L)	8.60 ± 4.02
HbA_1c_ (%)	9.06 ± 2.39
Alb (g/L)	35.82 ± 5.29
TC (mmol/L)	4.45 ± 1.29
LDL-C (mmol/L)	2.70 ± 1.00
HDL-C (mmol/L)	1.02 ± 0.30
**Proteinuria**	
Microalbuminuria (%)	1944 (21.8%)
Macroalbuminuria (%)	1542 (17.3%)
Uric Acid (umol/L)	313.22 ± 102.10
eGFR (mL/(min 1.73 m^2^))	124.68 ± 55.49
HOMA2-IR (≥ 1)	4310 (48.4%)
HOMA2-B (≥ 0.38)	4159 (46.7%)
**Complications**	
DR (%)	2742 (30.8%)
DPN (%)	4516 (50.7%)
DF (%)	685 (7.7%)
DN (%)	2962 (33.2%)
DK (%)	716 (8%)
CHD (%)	1865 (20.9%)
CVD (%)	1205 (13.5%)
HTN (%)	4974 (55.8%)
**Treatments**	
ACEI (%)	1559 (17.5%)
ARB (%)	2058 (23.1%)
Lipid-lowering agents (%)	6351 (71.3%)
OHA (%)	6313 (70.9%)
Insulin (%)	6258 (70.2%)

BMI, body mass index; WHR, waist-hip ratio; SBP, systolic blood pressure; DBP, diastolic blood pressure; Hb, hemoglobin; PLT, platelet; FBG, fasting blood-glucose; HbA_1C_, glycosylated hemoglobin; Alb, albumin; TC, total cholesterol; LDL-C, low-density lipoprotein-cholesterol; HDL-C, high-density lipoprotein-cholesterol; eGFR, estimated of glomerular filtration rate;HOMA2-B (homoeostatic model assessment 2-B) and HOMA2-IR (= homoeostatic model assessment 2-insulin resistance) were applied to estimate the function of β-cell and insulin resistance respectively, which calculated using fasting plasma glucose and c-peptide. DR, diabetic retinopathy; DPN, diabetic peripheral neuropathy; DF, diabetic foot; DN, diabetic nephropathy; DK, diabetic ketosis; CHD, coronary heart disease; CVD, cerebrovascular disease; HTN, hypertension; ACEI, angiotensin-converting enzyme inhibitor; ARB, angiotensin receptor blockers. OHA, oral hypoglycemic agents.

### Comparison of the clinical characteristics among the groups based on different numbers of first-degree relatives with diabetes

Based on the number of first-degree relatives with diabetes, the study was divided into 3 groups as follows: “No FHD” group referred to patients with no first-degree relatives with diabetes; the “One member with FHD” group referred to patients with only one first-degree relative with diabetes; the “ ≥ 2 members with FHD” group referred to patients with more than 2 first-degree relatives with diabetes. The following clinical characteristics were significantly different across the 3 groups: age, the proportion of men, DBP, the durations of diabetes, disease onset age, smoking and drinking habit, Hb levels, FBG, HbA_1c_ levels, Alb levels, TC levels, proteinuria, eGFR, HOMA2-IR and HOMA2-B, the prevalence of DR, DPN, DF, diabetic ketosis (DK), CHD, CVD, HTN, the use of lipid-lowering agents and OHA (*P* < 0.05). Other parameters, including BMI, WHR, SBP, PLT levels, LDL-C levels, HDL-C levels, uric acid levels, the prevalence of DN, and the use of ACEI, ARB and insulin, were not significantly different across groups (*P* ≥ 0.05) (see Table 2).

**Table 2:** Comparison of clinical characteristics among the 3 groups based on different number of FHD.

Basic characteristics	NO FHD (N = 6105)	One member with FHD (n = 1663)	≥ 2 members with FHD (n = 1141)	*P*
Age (years)	59.1 ± 12.5	54.3 ± 11.8	56.9 ± 9.7	< 0.001*
Men (%)	3373 (55.2%)	972 (58.4%)	602 (52.8%)	0.009*
BMI (kg/m^2^)	24.6 ± 3.7	24.6 ± 3.6	24.5 ± 3.4	0.512
WHR	0.94 ± 0.07	0.94 ± 0.07	0.94 ± 0.07	0.513
SBP (mmHg)	137.7 ± 20.8	136.8 ± 21.6	137.3 ± 20.5	0.304
DBP (mmHg)	80.2 ± 12.1	82.2 ± 12.3	80.4 ± 12.1	< 0.001*
Durations (years)	8.4 ± 6.7	8.3 ± 6.5	10.0 ± 6.4	< 0.001*
Disease onset age (years)	50.7 ± 11.6	46.1 ± 10.6	46.8 ± 9.6	< 0.001*
Smoking (%)	2025 (33.2%)	629 (37.8%)	415 (36.4%)	0.002*
Drinking (%)	1409 (23.0%)	453 (27.2%)	276 (24.2%)	0.004*
**Laboratory data**				
Hb (g/L)	125.6 ± 21.9	129.4 ± 21.6	126.5 ± 20.9	< 0.001*
PLT (*10^9^/L)	213.4 ± 83.2	214.8 ± 79.5	215.1 ± 76.1	0.73
FBG (mmol/L)	8.5 ± 4.0	8.9 ± 4.0	8.5 ± 3.9	0.001*
HbA_1c_ (%)	9.0 ± 2.4	9.3 ± 2.4	9.0 ± 2.2	< 0.001*
Alb (g/L)	35.7 ± 5.3	36.3 ± 5.2	35.8 ± 5.2	< 0.001*
TC (mmol/L)	4.4 ± 1.3	4.6 ± 1.4	4.5 ± 1.2	< 0.001*
LDL-C (mmol/L)	2.8 ± 1.0	2.9 ± 1.0	2.9 ± 1.0	0.169
HDL-C (mmol/L)	1.0 ± 0.3	1.0 ± 0.3	1.0 ± 0.3	0.172
**Proteinuria**				0.003*
Microalbuminuria (mg/24 h)	1366 (22.4%)	336 (20.2%)	242 (21.2%)	
Macroalbuminuria (mg/24 h)	1007 (16.5%)	297 (17.9%)	238 (20.9%)	
Uric acid (mmol/L)	314.5 ± 104.1	308.8 ± 97.0	312.9 ± 98.2	0.281
eGFR (mL/(min 1.73 m^2^))	122.6 ± 55.4	132.0 ± 56.2	125.2 ± 54.2	< 0.001*
HOMA2-IR (≥ 1)	3003 (49.2%)	795 (47.8%)	512 (44.9%)	0.003*
HOMA2-B (≥ 0.38)	2734 (44.8%)	871 (52.4%)	554 (48.6%)	< 0.001*
**Complications**				
DR (%)	1834 (30.0%)	499 (30%)	409 (35.8%)	< 0.001*
DPN (%)	3109 (50.9%)	780 (46.9%)	627 (55%)	< 0.001*
DF (%)	444 (7.3%)	134 (8.1%)	107 (9.4%)	0.041*
DN (%)	2021 (33.1%)	537 (32.3%)	737 (35.4%)	0.208
DK (%)	487 (8.0%)	162 (9.7%)	67 (5.9%)	0.001*
CHD (%)	1345 (22.0%)	295 (17.7%)	225 (19.7%)	< 0.001*
CVD (%)	864 (14.2%)	195 (11.7%)	146 (12.8%)	0.028*
HTN (%)	3465 (56.8%)	869 (52.3%)	640 (56.1%)	0.005*
**Treatments**				
ACEI (%)	1055 (17.3%)	310 (18.6%)	194 (17%)	0.387
ARB (%)	1424 (23.3%)	355 (21.3%)	280 (24.5%)	0.112
Lipid-lowering agents (%)	4298 (70.4%)	1205 (72.5%)	848 (74.3%)	0.014*
OHA (%)	4271 (70.0%)	1210 (72.8%)	832 (72.9%)	0.022*
Insulin (%)	4251 (69.6%)	1190 (71.6%)	817 (71.6%)	0.176

DM, diabetes mellitus; BMI, body mass index; WHR, waist-hip ratio; SBP, systolic blood pressure; DBP, diastolic blood pressure; Hb, hemoglobin; PLT, platelet; FBG, fasting blood-glucose; HbA_1C_, glycosylated hemoglobin; Alb, albumin; TC, total cholesterol; LDL-C, low-density lipoprotein-cholesterol; HDL-C, high-density lipoprotein-cholesterol; eGFR, estimated of glomerular filtration rate;HOMA2-B (homoeostatic model assessment 2-B) and HOMA2-IR (= homoeostatic model assessment 2-insulin resistance) were applied to estimate the function of β-cell and insulin resistance respectively, which calculated using fasting plasma glucose and c-peptide. DR, diabetic retinopathy; DPN, diabetic peripheral neuropathy; DF, diabetic foot; DN, diabetic nephropathy; DK, diabetic ketosis; CHD, coronary heart disease; CVD, cerebrovascular disease; HTN, hypertension; ACEI, angiotensin-converting enzyme inhibitor; ARB, angiotensin receptor blockers. OHA, oral hypoglycemic agents.

*Refers to the P < 0.05.

### Comparison of the clinical characteristics among the groups based on different types of first-degree relatives with diabetes

When comparing the clinical characteristics of the 8 groups, we found that the following parameters showed significant differences: age, the proportion of men, SBP, durations of the disease, disease onset age, smoking and drinking, Hb levels, FBG levels, HbA_1C_ levels, Alb levels, TC levels, LDL-C levels, proteinuria, uric acid levels, eGFR, HOMA2-B, DR, DPN, DN, DK, CHD, CVD, HTN, ARB, lipid-lowering agents, OHA, and insulin (*P* < 0.05). Other variables, including WHR, PLT levels, HDL-C levels, HOMA2-IR, DFCs and the use of ACEI, did not differ significantly across groups (*P* ≥ 0.05) (see Table 3).

**Table 3:** Comparison of clinical characteristics among the 8 groups based on different type of family history of diabetes.

Basic characteristics	NO FHD (N = 6105)	Father (N = 313)	Mother (N = 555)	Siblings (N = 1287)	Father and siblings (N = 137)	Mother and siblings (N = 354)	Both parents and siblings (N = 61)	Both parents (N = 97)	P
Age (years)	59.1 ± 12.5	46.4 ± 11.7	50.3 ± 10.0	60.4 ± 9.4	54.3 ± 9.6	55.8 ± 8.7	52.1 ± 7.5	48.1 ± 10.6	< 0.001*
Men (%)	3373 (55.2%)	212 (67.7%)	354 (63.8%)	646 (50.2%)	70 (51.1%)	196 (55.4%)	28 (45.9%)	68 (70.1%)	< 0.001*
BMI (kg/m^2^)	24.6 ± 3.7	25.3 ± 3.8	24.8 ± 3.8	24.3 ± 3.4	24.1 ± 3.0	24.3 ± 3.5	25.2 ± 4.0	25.5 ± 3.7	< 0.001*
WHR	0.9 ± 0.1	0.9 ± 0.1	0.9 ± 0.1	0.9 ± 0.1	0.9 ± 0.1	0.9 ± 0.1	0.9 ± 0.1	0.9 ± 0.1	0.123
SBP (mmHg)	137.7 ± 20.8	132.5 ± 18.3	136.9 ± 21.7	138.5 ± 21.9	137.7 ± 21.4	136.0 ± 20.4	135.8 ± 19.6	135.6 ± 17.9	0.001*
DBP (mmHg)	80.2 ± 12.1	82.6 ± 12.4	84.1 ± 12.3	80.3 ± 12.0	80.3 ± 12.9	80.4 ± 11.5	82.5 ± 13.3	84.1 ± 13.4	< 0.001*
Durations (years)	8.4 ± 6.7	6.7 ± 6.0	7.6 ± 6.1	10.0 ± 6.7	9.6 ± 6.4	10.1 ± 6.4	7.7 ± 6.1	6.7 ± 5.5	< 0.001*
Disease onset age (years)	50.7 ± 11.6	39.8 ± 9.9	42.7 ± 8.9	50.4 ± 9.7	44.6 ± 9.3	45.7 ± 8.9	44.4 ± 7.2	41.4 ± 9.7	< 0.001*
Smoking (%)	2025 (33.2%)	131 (41.9%)	234 (42.2%)	421 (32.7%)	52 (38.0%)	138 (39.0%)	20 (32.8%)	48 (49.5%)	< 0.001*
Drinking (%)	1409 (23.0%)	91 (29.0%)	174 (31.3%)	288 (22.4%)	34 (24.0%)	98 (27.7%)	15 (24.6%)	29 (29.9%)	< 0.001*
**Laboratory data**									
Hb (g/L)	125.6 ± 21.9	135.6 ± 21.2	132.7 ± 21.1	124.6 ± 20.1	124.6 ± 20.7	127.0 ± 19.6	129.5 ± 19.4	136.1 ± 21.7	< 0.001*
PLT (*10^9^/L)	213.4 ± 83.2	221.3 ± 91.1	216.7 ± 76.0	212.8 ± 76.3	222.2 ± 94.0	211.8 ± 71.4	216.6 ± 65.7	211.2 ± 74.7	0.604
FBG (mmol/L)	8.5 ± 4.0	9.6 ± 4.5	8.9 ± 4.0	8.7 ± 4.0	8.3 ± 4.0	8.3 ± 3.6	8.9 ± 4.1	8.9 ± 3.8	< 0.001*
HbA_1_c (%)	9.0 ± 2.4	9.6 ± 2.5	9.3 ± 2.4	9.1 ± 2.3	8.9 ± 2.2	8.9 ± 2.2	8.6 ± 1.8	9.1 ± 2.1	0.004*
Alb (g/L)	35.7 ± 5.3	37.2 ± 4.8	36.7 ± 5.2	35.6 ± 5.2	36.1 ± 5.3	35.2 ± 5.5	36.7 ± 4.5	38.1 ± 4.1	< 0.001*
TC (mmol/L)	4.4 ± 1.3	4.7 ± 1.4	4.7 ± 1.4	4.5 ± 1.3	4.7 ± 1.2	4.5 ± 1.3	4.5 ± 1.2	4.5 ± 1.2	< 0.001*
LDL-C (mmol/L)	2.8 ± 1.0	3.0 ± 1.1	2.9 ± 1.0	2.8 ± 1.0	3.0 ± 1.0	2.9 ± 1.1	2.8 ± 1.0	2.7 ± 1.0	< 0.001*
HDL-C (mmol/L)	1.0 ± 0.3	1.0 ± 0.3	1.0 ± 0.3	1.0 ± 0.3	1.0 ± 0.3	1.0 ± 0.3	1.0 ± 0.2	1.0 ± 0.3	0.078
**Proteinuria**									0.002*
Microalbuminuria (mg/24 h)	1366 (22.4%)	67 (21.4%)	106 (19.1%)	265 (20.6%)	23 (16.8%)	79 (22.3%)	14 (23.0%)	24 (24.7%)	
Macroalbuminuria (mg/24 h)	1007 (16.5%)	52 (16.6%)	100 (18.0%)	247 (19.2%)	39 (28.5%)	79 (22.3%)	6 (9.8%)	12 (9.8%)	
Uric acid (mmol/L)	314.5 ± 104.1	322.4 ± 99.9	312.2 ± 92.6	307.6 ± 100.4	299.9 ± 90.1	306.6 ± 96.3	302.9 ± 88.3	333.8 ± 90.9	0.009*
eGFR (mL/(min 1.73 m^2^))	122.6 ± 55.4	146.8 ± 55.2	139.6 ± 56.7	118.2 ± 52.0	131.6 ± 65.6	128.4 ± 54.8	145.0 ± 53.1	147.8 ± 50.7	< 0.001*
HOMA2-IR (≥ 1)	3003 (49.2%)	155 (49.5%)	257 (46.3%)	600 (46.6%)	63 (46.0%)	153 (43.2%)	29 (47.5%)	50 (51.5%)	0.061
HOMA2-B (≥ 0.38)	2734 (44.8%)	183 (58.5%)	286 (51.5%)	641 (49.8%)	58 (42.3%)	172 (48.6%)	31 (50.8%)	54 (55.7%)	< 0.001*
**Complications**									
DR (%)	1834 (30.0%)	71 (22.7%)	157 (28.3%)	468 (36.4%)	47 (34.3%)	131 (37%)	14 (23.0%)	20 (20.6%)	< 0.001*
DPN (%)	3109 (50.9%)	101 (32.3%)	237 (42.7%)	755 (58.7%)	65 (47.4%)	192 (54.2%)	30 (49.2%)	27 (27.8%)	< 0.001*
DF (%)	444 (7.3%)	20 (6.4%)	45 (8.1%)	121 (9.4%)	12 (8.8%)	31 (8.8%)	5 (8.2%)	7 (7.2%)	0.288
DN (%)	2021 (33.1%)	88 (28.1%)	176 (31.7%)	456 (35.4%)	58 (42.3%)	120 (33.9%)	16 (26.2%)	27 (27.8%)	0.037*
DK (%)	487 (8.0%)	45 (14.4%)	53 (9.5%)	86 (6.7%)	11 (8.0%)	23 (6.5%)	4 (6.6%)	7 (7.2%)	0.001*
CHD (%)	1345 (22.0%)	37 (11.8%)	84 (15.1%)	296 (23.0%)	17 (12.4%)	70 (19.8%)	8 (13.1%)	8 (8.2%)	< 0.001*
CVD (%)	864 (14.2%)	23 (7.3%)	45 (8.1%)	206 (16.0%)	11 (8.0%)	41 (11.6%)	9 (14.8%)	6 (6.2%)	< 0.001*
HTN (%)	3465 (56.8%)	132 (42.2%)	270 (48.6%)	772 (60%)	75 (54.7%)	189 (53.3%)	27 (44.3%)	44 (45.4%)	< 0.001*
**Treatments**									
ACEI (%)	1055 (17.3%)	62 (19.8%)	88 (15.9%)	258 (20%)	20 (14.6%)	54 (15.3%)	7 (11.5%)	15 (15.5%)	0.1
ARB (%)	1424 (23.3%)	47 (15.0%)	128 (23.1%)	299 (23.2%)	33 (24.1%)	91 (25.7%)	17 (27.9%)	20 (20.6%)	0.047*
Lipid-lowering agents (%)	4298 (70.4%)	217 (69.3%)	396 (71.4%)	975 (75.8%)	96 (70.1%)	255 (72.0%)	47 (77.0%)	67 (69.1%)	0.018*
OHA (%)	4271 (70.0%)	245 (78.3%)	408 (73.5%)	891 (69.2%)	105 (76.6%)	262 (74.0%)	52 (85.2%)	79 (81.4%)	< 0.001*
Insulin (%)	4251 (69.6%)	222 (70.9%)	382 (68.8%)	947 (73.6%)	104 (75.9%)	254 (71.8%)	36 (59.0%)	62 (63.9%)	0.02*

BMI, body mass index; WHR, waist-hip ratio; SBP, systolic blood pressure; DBP, diastolic blood pressure; Hb, hemoglobin; PLT, platelet; FBG, fasting blood-glucose; HbA_1C_, glycosylated hemoglobin; Alb, albumin; TC, total cholesterol; LDL-C, low-density lipoprotein-cholesterol; HDL-C, high-density lipoprotein-cholesterol;TG, triglyceride; eGFR, estimated of glomerular filtration rate; HOMA2-B ,homoeostatic model assessment 2-B; HOMA2-IR, homoeostatic model assessment 2-insulin resistance; DR, diabetic retinopathy; DPN, diabetic peripheral neuropathy; DF, diabetic foot; DN, diabetic nephropathy; DK, diabetic ketosis; CHD, coronary heart disease; CVD, cerebrovascular disease; HTN, hypertension; ACEI, angiotensin-converting enzyme inhibitor; ARB, angiotensin receptor blockers. OHA, oral hypoglycemic agents.

*Refers to the *P* < 0.05.

### The association between DFCs and clinical characteristics based on different numbers and types of first-degree relatives with diabetes

The multivariable regression analysis showed DFCs was positively associated with sex (OR = 1.596, 95%CI 1.249–2.039), having one first-degree family member with diabetes (OR = 1.377, 95%CI 1.087–1.744), having more than one family first-degree member with diabetes (OR = 1.402, 95%CI 1.079–1.822), PLT levels higher than 300 * 10^9^/L (OR = 3.158, 95%CI 1.800–5.540), DPN (OR = 2.696, 95%CI 2.159–3.366), the using of lipid-lowering agents (OR = 1.721, 95%CI 1.369–2.164) and the use of insulin (OR = 1.650, 95%CI 1.248–2.182). BMI (OR = 0.787, 95%CI 0.647–0.956), Hb levels (OR = 0.416, 95%CI 0.332–0.522), HDL-C levels (OR = 0.660, 95%CI:0.537–0.811), Alb levels (OR = 0.585, 95%CI 0.420–0.816), and OHA (OR = 0.529, 95%CI 0.431–0.649) were negatively associated with DFCs. Other variables, including DBP, duration, disease onset age, smoking, HbA_1c_ levels eGFR, and HOMA2-IR, were not significantly associated with DFCs (*P* ≥ 0.05).

When the different types of first-degree relatives with diabetes was entered into the analysis instead of the number of first-degree relatives with diabetes, the results revealed that only having a mother with a history diabetes was positively associated with diabetes (OR = 1.484, 95%CI 1.022–2.154); other types, including a father, siblings, siblings and a mother, siblings and a father, both parents and siblings, both parents, displayed no significance (*P* > 0.05). Regarding other variables, such as BMI, Hb levels, PLT levels, DPN, and OHA, similar results were observed with the abovementioned variables in Table 4 (see Table 5).

**Table 4:** Multi-variable regression based on diabetic foot and clinical characteristics, different number of family history of diabetes.

	B	Wald	*P*	OR (95%CI)
Sex	0.467	13.949	< 0.001*	1.596 (1.249–2.039)
DBP	− 0.166	2.916	0.088	0.847 (0.700–1.025)
Durations	− 0.143	1.890	0.169	0.867 (0.707–1.063)
Disease onset age	0.004	0.002	0.967	1.004 (0.828–1.218)
Smoking	− 0.014	0.014	0.907	0.986 (0.777–1.251)
BMI	− 0.240	5.842	0.016*	0.787 (0.647–0.956)
**Family number with diabetes**				
1	0.320	7.056	0.008*	1.377 (1.087–1.744)
≥ 2	0.338	6.388	0.011*	1.402 (1.079–1.822)
Hb	− 0.876	57.284	< 0.001*	0.416 (0.332–0.522)
PLT (100–300)	0.410	2.259	0.133	1.507 (0.883–2.574)
PLT (> 300)	1.150	16.074	< 0.001*	3.158 (1.800–5.540)
TC	− 0.265	2.531	0.112	0.767 (0.553–1.064)
LDL-C	− 0.141	1.032	0.310	0.869 (0.662–1.140)
HDL-C	− 0.416	15.665	< 0.001*	0.660 (0.537–0.811)
Alb	− 0.536	9.947	0.002*	0.585 (0.420–0.816)
HbA_1c_	0.132	1.662	0.197	1.142 (0.933–1.396)
eGFR	− 0.003	0.001	0.981	0.997 (0.790–1.259)
HOMA2-IR	− 0.067	0.427	0.513	0.935 (0.765–1.143)
DPN	0.992	76.587	< 0.001*	2.696 (2.159–3.366)
Lipid-lowering agents	0.543	21.572	< 0.001*	1.721 (1.369–2.164)
OHA	− 0.637	37.194	< 0.001*	0.529 (0.431–0.649)
Insulin	0.501	12.365	< 0.001*	1.650 (1.248–2.182)
Constant	− 3.285	85.729	< 0.001	0.037

DBP, diastolic blood pressure; BMI, body mass index; Hb, hemoglobin; PLT, platelet; TC, total cholesterol; LDL-C, low-density lipoprotein-cholesterol; HDL-C, high-density lipoprotein-cholesterol; Alb, albumin; HbA_1C_, glycosylated hemoglobin; eGFR, estimated of glomerular filtration rate; HOMA2-IR, homoeostatic model assessment 2-insulin resistance; DPN, diabetic peripheral neuropathy; OHA, oral hypoglycemic agents.

*Refers to the *P* < 0.05.

**Table 5:** Multi-variable regression based on diabetic foot and clinical characteristics, different types of family history of diabetes.

	B	Wald	*P*	OR (95% CI)
Sex	0.465	13.784	< 0.001*	1.592 (1.246–2.035)
DBP	− 0.166	2.894	0.089	0.847 (0.700–1.026)
Durations	− 0.135	1.674	0.196	0.874 (0.713–1.072)
Disease onset age	0.022	0.047	0.829	1.022 (0.840–1.243)
Smoking	− 0.016	0.017	0.897	0.984 (0.775–1.250)
BMI	− 0.244	6.006	0.014*	0.784 (0.645–0.952)
**FHD**				
Father	0.472	3.193	0.074	1.603 (0.955–2.690)
Mother	0.395	4.306	0.038*	1.484 (1.022–2.154)
Siblings	0.238	3.462	0.063	1.268 (0.987–1.630)
Father and siblings	0.326	0.769	0.381	1.385 (0.669–2.870)
Mother and siblings	0.415	3.771	0.052	1.514 (0.996–2.302)
Both parents and siblings	0.511	0.878	0.349	1.668 (0.572–4.861)
Both parents	0.551	1.446	0.229	1.735 (0.707–4.256)
Hb	− 0.877	57.145	< 0.001*	0.416 (0.332–0.522)
PLT (100–300)	0.408	2.231	0.135	1.504 (0.880–2.568)
PLT (> 300)	1.151	16.118	< 0.001*	3.162 (1.803–5.548)
TC	− 0.268	2.573	0.109	0.765 (0.552–1.061)
LDL	− 0.141	1.034	0.309	0.869 (0.662–1.140)
HDL	− 0.417	15.691	< 0.001*	0.659 (0.536–0.810)
Alb	− 0.539	10.045	0.002*	0.583 (0.418–0.814)
HbA_1c_	0.134	1.701	0.192	1.143 (0.935–1.399)
eGFR	− 0.008	0.004	0.949	0.992 (0.786–1.253)
HOMA2-IR	− 0.065	0.404	0.525	0.937 (0.766–1.146)
DPN	1.000	77.367	< 0.001*	2.719 (2.176–3.398)
Lipid-lowering agents	0.546	21.759	< 0.001*	1.726 (1.372–2.171)
OHA	− 0.643	37.589	< 0.001*	0.526 (.428–.646)
Insulin	0.500	12.288	< 0.001*	1.648 (1.246–2.179)
Constant	− 3.297	86.203	< 0.001*	0.037

DBP, diastolic blood pressure; BMI, body mass index; FHD, family history of diabetes; Hb, hemoglobin; PLT, platelet; TC, total cholesterol; LDL-C, low-density lipoprotein-cholesterol; HDL-C, high-density lipoprotein-cholesterol; Alb, albumin; HbA_1C_, glycosylated hemoglobin; eGFR, estimated of glomerular filtration rate; HOMA2-IR, homoeostatic model assessment 2-insulin resistance; DPN, diabetic peripheral neuropathy; OHA, oral hypoglycemic agents.

*Refers to the *P* < 0.05.

## Discussion

In this study, we found that DFCs among T2DM patients were associated with FHD, including both the number and type of first-degree family members with diabetes. However, among the different types of first-degree family members with diabetes, only the patients with a mother with a history of diabetes showed a statistical association with DFCs. In addition to FHD, other factors, including gender, BMI, PLT levels, Hb levels, Alb levels, HDL-C levels, DPN, and the use of lipid-lowering agents, OHA, and insulin, were also associated with DFCs.

The management of diabetes is an important part of public health policy. However, it is not going well, especially the management of diabetic complications, such as DFCs^21,22^. A previous study reported that the overall number of amputations is still high^21^. Thus, identifying the risk factors for DFCs among diabetes patients is important. Previous studies have revealed that many factors are associated with DFCs, such as smoking, sex, FBS, and HbA_1c_ levels^23–25^. In our study, we observed a strong relationship between FHD and DFCs, especially of the different numbers of relatives with diabetes, in which the number of patients with one and more than 1 relative with diabetes led to 1.377- and 1.402-fold increases in the risk of DFCs, respectively, compared with those without FHD.

FHD has been considered a reflection of both genetic effects and environmental effects^26^. Regarding the genetic effects, with the rapid advancement in currently emerging genetic methods, such as genome-wide association studies (GWAS), a wide variety of candidate genes associated with T2DM have been identified^27^. The genetic risk score (GRS) has been reported to be associated with FHD^28,29^. A study investigated whether the GRS value was associated with parental numbers with FHD, indicating an important role of genes and FHD^28^. Similarly, genetic factors may be vital in DFCs, and diabetes-related complications have been reported to have a phenomenon of family clustering in type 1 diabetes^30^ and type 2 diabetes^31^. Genetic and ethnic factors play an important role in not only DPN but also DFCs^32^. Several studies have identified that some genetic variants may be potential risk factors for DFCs, such as the polymorphisms of MAPK14^33^, TNFRSF11B^34^, MCP1^35^, VEGF^36,37^, TNF-α^38^, and osteoprotegerin gene^39^. In addition, one genome-wide association study conducted by Meng et al.^33^, which included 699 diabetic foot ulcers and 2695 controls, indicated that the single‐nucleotide polymorphism rs80028505 of MAPK14 was a risk factor for diabetic foot ulcers. Another study aimed to investigate the relationship between VEGF gene polymorphisms and DFCs in a Chinese population showed that the VEGF gene could be a susceptible gene of DFCs^36^. Another study found that polymorphisms in cytokine/chemokine genes had a close association with amputation and severe infection in individuals with DFCs^38^. Therefore, we conjectured from the abovementioned studies that the higher prevalence of DFCs in patients with FHD may be due to genetic impact.

A study to investigate the association between different relatives with diabetes and the prevalence of T1DM, LADA and T2DM found that any first-degree member with diabetes, such as a father with diabetes, a mother with diabetes, or siblings with diabetes, would increase the prevalence of LADA and T2DM^14^. When we explored the relationship between different types of FHD and DFCs, the results suggested that only having a mother with a history of diabetes was associated with DFCs after adjusting for some usual confounders of DFCs; other types of diabetes showed no statistical association. The mechanism remains unknown. Some studies observed maternal transmission in T2DM^40,41^. The hypotheses attributed the maternal transmission to the distinctly maternal genetic and environmental impact^42^. Thus, we speculated that maternal transmission likely existed in DFC individuals and that the high prevalence of DFCs in patients with a mother history of diabetes may be caused by maternal transmission. Another possible explanation was that environmental factors, such as smoking and drinking^23,43,44^ and BMI^45^, also contributed to DFCs. In our study, participants in mother history seemingly had unhealthy lifestyles with worse weight management and glycemic control and a higher percentage of smokers and drinkers (Table 3). Additionally, family lifestyles, including eating habits, are mainly derived from mothers, and mothers’ history of diabetes may be easily inclined to live with unhealthy lifestyles, leading to a higher prevalence of DFCs.

Our study found that male gender was a risk factor for DFCs. A study aimed to investigate 88 potential risk factors for amputation in DFC patients implied that male gender increased the likelihood of amputation by eightfold compared to female gender^46^. On the one hand, males tend to have higher foot pressure and a higher prevalence of peripheral insensate neuropathy, which may contribute to DFCs^47,48^. On the other hand, females may be inclined to be more self-managed and self-caring and show an active attitude towards foot care; however, males are more likely to express fear, inactive attitudes and an uncooperative manner in behavior^49,50^.

PLT levels were also a risk factor for DFCs. Similarly, a study observed a positive association between amputation and PLT levels in diabetic forefoot ulcers^46^. PLT hyperreactivity, mainly caused by hyperglycemia, metabolic disturbance and insulin resistance, plays an important role in the prothrombotic state of diabetes^51^. A prothrombotic state may result in atherogenesis attributable to the impaired endothelium or ruptured plaque by abnormal adhesion and aggregation of PLT levels^52^. Furthermore, inflammation has an association with endothelial injury and regulates platelet action-associated proteins^53^. Atherogenesis and inflammation may contribute to the occurrence of PAD, which has been demonstrated to be a risk factor for DFCs^7,54^.

The relationship between BMI and DFCs remains controversial^23,46,55^. On the one hand, a study investigated whether BMI had no association with diabetic foot ulcers^23^. On the other hand, another study showed a positive association between BMI and DFCs^8^, contrary to our results. This may be caused by racial differences. Dyslipidemia, such as the decreased level of HDL and increased level of triglycerides, has been proven to be related to DFCs^44,56^, consistent with our study. Due to the negative association between DFCs and HDL, it was easy to comprehend the higher percentage of lipid-lowering agent use in DFC patients. Moreover, one multicentric cross-sectional study revealed that insulin was positively associated with DFCs^57^, consistent with our findings that DFC patients had a higher proportion of insulin**.**

Several limitations were observed in the study. First, it was not clear whether the first-degree family members with diabetes had type 1 diabetes or type 2 diabetes. Second, this was a cross-sectional and single-center observation, and more prospective and multicenter studies should be conducted.

In conclusion, DFCs were associated with different numbers and types of family members with diabetes. This association reveals the importance of genetic and environmental factors in DFCs and highlights the importance of adding FHD to public health strategies targeting detecting and preventing the disease.

## Supplementary information

Supplementary Information.

## Data Availability

The data included in the manuscript are available.
