# Supplementary material for: Family history of diabetes is associated with diabetic foot complications in type 2 diabetes
Source: Sci Rep. 2020 Oct 13;10:17056. doi: 10.1038/s41598-020-74071-3 (PMC7555504; doi:10.1038/s41598-020-74071-3)
Supplement: Supplementary file 1 — Supplementary Information. [file 41598_2020_74071_MOESM1_ESM.docx]

**Family History of Diabetes is Associated with Diabetic Foot Complications in Type 2 Diabetes**

Xiao-fen Xiong,^1^ Ling Wei,^1^ Ying Xiao,^1^ Ya-Chun Han, ^1^ Jinfei Yang, ^1^ Hao Zhao, ^1^ Ming Yang, ^1^ Lin Sun*^1^

**Supporting information**

Table 1 Flow Chart of the study

Diagnosed with Diabetes between January 2013 and November 2018 (N=11800)

1.Type 1 Diabetes (N=957)

2.Latent Autoimmune Diabetes in Adults (N=365)

Diagnosed with Type 2 Diabetes (N=10478)

1.Age＜18y (N=517)

2.The patients in pregnancy or lactation (N=365)

3. cancers (N=205)

4. Secondary disease resulted in elevated blood glucose(N=239)

5. patients with missing values of FHD and DFCs(N=243)

The Whole Cohort (N=8909)

Table 2 Value Assignment of the Clinical Characteristics in logistic regression

| Clinical Characteristics | Value Assignment |
| --- | --- |
| Male | “0” refers to “female”; “1” refers to “male” |
| SBP | “0” refers to “＜130mmHg”; “1” refers to “≥130mmHg” |
| DBP | “0” refers to “＜80mmHg”; “1” refers to “≥80mmHg” |
| Durations | “0” refers to “＜8”; “1” refers to “≥8” |
| Disease onset age | “0” refers to “＜49”; “1” refers to “≥49” |
| Smoking | “0” refers to “no smoking”; “1” refers to “smoking” |
| Drinking | “0” refers to “no drinking”; “1” refers to “drinking” |
| BMI | “0” refers to “＜24”; “1” refers to “≥24” |
| WHR | “0” refers to “＜0.94”; “1” refers to “≥0.94” |
| FHD | “0” refers to “no FHD”; “1” refers to “father history”; “2” refers to “mother history”;“3” refers to “sibling history”; “4” refers to “father and sibling history”; “5” refers to “mother and sibling history”; “6” refers to “both parents and sibling history”; “7” refers to “both parents history” |
| Family number with diabetes | “0” refers to “no FHD”; “1” refers to “one family member with diabetes”; “2” refers to “more than one family member with diabetes” |
| Hb | “0” refers to “＜110”; “1” refers to “≥110” |
| PLT | “0” refers to “＜100”; “1” refers to “100-300”; “3” refers to “≥300” |
| LDL-C | “0” refers to “＜3.12”; “1” refers to “≥3.12” |
| TC | “0” refers to “＜5.2”; “1” refers to “≥5.2” |
| HDL-C | “0” refers to “＜1.04”; “1” refers to “≥1.04” |
| Alb | “0” refers to “＜40”; “1” refers to “≥40” |
| Glu | “0” refers to “＜8.6”; “1” refers to “≥8.6” |
| HbA_1c_ | “0” refers to “＜9”; “1” refers to “≥9” |
| HOMA2-IR | “0” refers to “＜1”; “1” refers to “≥1” |
| HOMA2-B | “0” refers to “＜38”; “1” refers to “≥38” |
| eGFR | “0” refers to “＜90”; “1” refers to “≥90” |
| Uric acid | “0” refers to “＜357”; “1” refers to “≥357” |
| ACEI | “0” refers to “no using ACEI”; “1” refers to “using ACEI” |
| ARB | “0” refers to “no using ARB”; “1” refers to “using ARB” |
| Lipid-lowering agents | “0” refers to “no using lipid-lowering agents”; “1” refers to “using lipid-lowering agents” |
| OHA | “0” refers to “no using OHA”; “1” refers to “using OHA” |
| Insulin | “0” refers to “no using insulin”; “1” refers to “using insulin” |

SBP, systolic blood pressure; DBP, diastolic blood pressure; BMI, body mass index; WHR, [waist-hip ratio](C:/Program%20Files%20(x86)/Youdao/Dict/7.5.2.0/resultui/dict/javascript:;); FHD, family history of diabetes; Hb, hemoglobin; PLT, platelet ; LDL-C, [low-density lipoprotein](C:/Program%20Files%20(x86)/Youdao/Dict/7.5.2.0/resultui/dict/javascript:;)-cholesterol; TC, total cholesterol; HDL-C, [high-density lipoprotein](C:/Program%20Files%20(x86)/Youdao/Dict/7.5.2.0/resultui/dict/javascript:;)-cholesterol; Alb, albumin; FBG, fasting blood-glucose ; HbA_1C_，glycosylated hemoglobin; HOMA2-B, homoeostatic model assessment 2-B; HOMA2-IR, homoeostatic model assessment 2-insulin resistance; eGFR, estimated of glomerular filtration rate; ACEI, angiotensin-converting enzyme inhibitor; ARB, angiotensin receptor blockers. OHA, oral hypoglycemic agents.

Table 3 Univariate Regression Based on Diabetic Foot and Clinical Characteristics

| Clinical Characteristics | B | Wald | P | OR (95%CI) |
| --- | --- | --- | --- | --- |
| Male | 0.325 | 15.665 | ＜0.001* | 1.384(1.178-1.625) |
| SBP | 0.004 | 0.003 | 0.957 | 1.004(0.854-1.181) |
| DBP | -0.358 | 19.894 | ＜0.001* | 0.699(0.597-0.818) |
| Durations | 0.444 | 29.408 | ＜0.001* | 1.559(1.328-1.831) |
| Disease onset age | 0.038 | 0.223 | 0.637 | 1.038(0.888-1.214) |
| Smoking | 0.242 | 8.809 | 0.003* | 1.274(1.086-1.495) |
| Drinking | 0.145 | 2.558 | 0.11 | 1.156(0.968-1.380) |
| BMI | -0.429 | 25.421 | ＜0.001* | 0.651(0.551-0.77) |
| WHR | -0.011 | 0.017 | 0.896 | 0.989(0.835-1.171) |
| FHD |  |  |  |  |
| Father | -0.139 | 0.346 | 0.557 | 0.87(0.548-1.383) |
| Mother | 0.118 | 0.521 | 0.47 | 1.125(0.817-1.549) |
| Siblings | 0.28 | 6.787 | 0.009* | 1.323(1.072-1.633) |
| Father and siblings | 0.202 | 0.436 | 0.509 | 1.224(0.672-2.231) |
| Mother and siblings | 0.202 | 1.079 | 0.299 | 1.224(0.836-1.791) |
| Both parents and siblings | 0.13 | 0.076 | 0.782 | 1.138(0.454-2.856) |
| Both parents | -0.008 | 0.000 | 0.983 | 0.992(0.457-2.153) |
| number of family history of diabetes |  |  |  |  |
| One family member with diabetes | 0.111 | 1.168 | 0.28 | 1.117 (0.914-1.367) |
| ≥2 family member with diabetes | 0.277 | 6.029 | 0.014 | 1.319 (1.058-1.646) |
| Hb | -0.03 | 295.166 | 0.000* | 0.97(0.967-0.974) |
| PLT (100-300) | 0.13 | 0.292 | 0.589 | 1.139(0.71-1.826) |
| PLT (＞300) | 1.368 | 30.07 | ＜0.001* | 3.927(2.408-6.403) |
| LDL-C | -0.468 | 25.217 | ＜0.001* | 0.626(0.522-0.752) |
| TC | -0.612 | 28.75 | ＜0.001* | 0.542(0.434-0.678) |
| HDL-C | -0.57 | 42.623 | ＜0.001* | 0.565(0.476-0.671) |
| Alb | -1.286 | 68.727 | ＜0.001* | 0.276(0.204-0.375) |
| FBG | -0.006 | 0.006 | 0.938 | 0.994(0.846-1.167) |
| HbA_1c_ | 0.022 | 0.075 | 0.784 | 1.023(0.871-1.200) |
| HOMA2-IR | -0.236 | 8.292 | 0.004* | 0.79(0.673-0.927) |
| HOMA2-B | 0.022 | 0.075 | 0.784 | 1.023 (0.871-1.200) |
| Uric acid | -0.041 | 0.219 | 0.64 | 0.96(0.807-1.141) |
| eGFR | -0.635 | 58.515 | ＜0.001* | 0.53(0.45-0.624) |
| ACEI | -0.054 | 0.26 | 0.61 | 0.947(0.769-1.167) |
| ARB | 0.119 | 1.665 | 0.197 | 1.126(0.94-1.348) |
| Lipid-lowering agents | 0.327 | 12.074 | 0.001* | 1.387(1.153-1.667) |
| OHA | -1.165 | 208.875 | ＜0.001* | 0.312(0.266-0.365) |
| Insulin | 1.15 | 95.329 | ＜0.001* | 3.157(2.506-3.976) |

SBP, systolic blood pressure; DBP, diastolic blood pressure; BMI, body mass index; WHR, [waist-hip ratio](C:/Program%20Files%20(x86)/Youdao/Dict/7.5.2.0/resultui/dict/javascript:;); FHD, family history of diabetes; Hb, hemoglobin; PLT, platelet ; LDL-C, [low-density lipoprotein](C:/Program%20Files%20(x86)/Youdao/Dict/7.5.2.0/resultui/dict/javascript:;)-cholesterol; TC, total cholesterol; HDL-C, [high-density lipoprotein](C:/Program%20Files%20(x86)/Youdao/Dict/7.5.2.0/resultui/dict/javascript:;)-cholesterol; Alb, albumin; FBG, fasting blood-glucose ; HbA_1C_，glycosylated hemoglobin; HOMA2-B, homoeostatic model assessment 2-B; HOMA2-IR, homoeostatic model assessment 2-insulin resistance; eGFR, estimated of glomerular filtration rate; ACEI, angiotensin-converting enzyme inhibitor; ARB, angiotensin receptor blockers. OHA, oral hypoglycemic agents. * refers to the *P*＜0.05.
